# Trends in the incidence of major birth defects after assisted reproductive technologies in Lombardy Region, Northern Italy

**DOI:** 10.1007/s10815-023-02732-z

**Published:** 2023-02-10

**Authors:** Giulia Galati, Giovanna Esposito, Edgardo Somigliana, Ludovico Muzii, Matteo Franchi, Giovanni Corrao, Fabio Parazzini

**Affiliations:** 1 Department of Maternal and Child Health and Urological Sciences, Sapienza University of Rome, Rome, Italy; 2 Department of Clinical Sciences and Community Health, University of Milan, Milan, Italy; 3 Department of Obstetrics, Gynecology and Neonatology, Fondazione IRCCS Ca’ Granda Ospedale Maggiore Policlinico, Milan, Italy; 4 Laboratory of Healthcare Research & Pharmacoepidemiology, Department of Statistics and Quantitative Methods, University of Milano-Bicocca, Milan, Italy; 5 National Centre for Healthcare Research and Pharmacoepidemiology, Milan, Italy

**Keywords:** Birth defects, Congenital malformation, ICSI, IVF, Multiple pregnancy

## Abstract

**Purpose:**

The incidence of birth defects is increased in children born after assisted reproductive technologies (ART). However, changes in ART over time could influence this incidence. To investigate this issue, we present the frequency and trends of birth defects in ART and natural pregnancies in Lombardy, Northern Italy, during the period 2014–2020.

**Methods:**

This is a population-based study using automated system of healthcare utilization (HCU) databases. ART pregnancies included only those obtained with conventional IVF or ICSI. After identifying the total number of deliveries, the rate of newborns with birth defects was compared between natural and ART pregnancies. A logistic regression model was used to calculate the adjusted odd ratio (OR). Analyses were repeated for every calendar year.

**Results:**

Overall, 508,421 deliveries were identified, of which 14,067 (2.8%) were achieved after IVF-ICSI. A decreasing trend in birth defects over time was highlighted. The adjusted OR dropped from 1.40 (95%CI: 1.03–1.91) in 2014 to 0.92 (95%CI: 0.69–1.22) in 2020. During the study period, a significant reduction in multiple pregnancy and in the ratio of ICSI to conventional IVF was also observed, explaining at least in part the positive trend observed for birth defects.

**Conclusion:**

The increased risk of birth defects in children born after IVF-ICSI is not steady over time. A decline of this risk emerged in our region. Policy changes in ART may explain this beneficial effect.

**Supplementary Information:**

The online version contains supplementary material available at 10.1007/s10815-023-02732-z.

## Introduction

Birth defects are a major public health issue since they represent the primary cause of infant mortality and lifetime disability [1]. Overall, around 3 to 5% of live births are affected by congenital abnormalities [2].

The link between assisted reproductive techniques (ART) and birth defects has been extensively studied. Concerns emerged with the advent of in vitro fertilization (IVF) and were re-fueled when Intra-cytoplasmatic sperm injection (ICSI) was introduced. This alarmism, however, progressively tempered over time [3]. The most recent meta-analysis included 46 studies and reported a significant but modest increased risk of birth defects for both conventional IVF and ICSI, the relative risks (RR) being 1.25 (95% confidence interval-CI: 1.12–1.40) and 1.29 (95%CI: 1.14–1.45), respectively [4]. Urogenital and circulatory systems appear to be the most frequently involved but data is not univocal [5–7]. Even if multiple pregnancies are an independent risk factor for birth defects, their detrimental effect in ART is only additive [8]. When compared to natural twins, the relative risk of congenital malformations in ART twin pregnancies is increased in the same manner as for singletons [9, 10]. Furthermore, an independent detrimental role of ICSI was claimed, but the evidence is controversial [11]. The latest available meta-analysis on all types of malformations reported a RR with ICSI of 1.05 (95%CI: 0.91–1.20) [12], but a subsequent meta-analysis exclusively focusing on genitourinary malformation highlighted a RR of 1.27 (95%CI: 1.02–1.59) [13].

The determinants of the association between ART and birth defects are not fully elucidated. The underlying infertility was advocated to play a role, supporting the view that the reported increased risk would be a mere association rather than due to a causal link, but the evidence is not definitive [8, 14]. Even if infertile women are at increased risk of birth defects per se, one cannot rule out an additional harmful effect of ART. To note, a causal relation is likely for some rare but troublesome imprinting disorders such as Beckwith–Wiedemann syndrome [15].

Overall, even if available meta-analyses on birth defects of ART babies include thousands of newborns, the argument cannot be deemed solved. Of further relevance here is that ART techniques are in continuous evolution. The most striking events occurring over the last decade include the increase of elective single embryo transfer, the diffusion of extended culture, and the growing use of frozen transfers. In addition, there is an extreme heterogeneity among centers in terms of laboratory conditions and clinical policies, and changes are rapid [16, 17]. Overall, it cannot be ruled out that these changes and this variability in ART could influence the incidence of birth defects [18, 19]

For this reason, we deemed of interest evaluating the possible modifications in the rate of birth defects in children born after ART over time. To this aim, we present the frequency and trends of birth defects in ART and natural pregnancies during the period 2014–2020 in Lombardy.

## Materials and methods

We analyzed the automated system of healthcare utilization (HCU) databases from Lombardy, the largest region in Northern Italy accounting for about 10 million inhabitants, that collects information about all health services reimbursable by the National Health Service (NHS). The process of selection is reported in detail elsewhere and herein briefly summarized [20, 21]. According to Italian law, analysis of anonymous administrative database does not require ethics committee approval.

We identified all deliveries in Lombardy that occurred between 1 January 2014 and 31 December 2020 from women beneficiaries of the NHS using the standard discharge form [scheda di dimissione ospedaliera (SDO)] and the certificate of delivery assistance [Certificato di assistenza al parto (CedAP)]. Exclusion criteria were the following: (i) deliveries which did not match to a SDO related to childbirth coded according to the International classification of disease, ninth revision (ICD-9) and/or the diagnosis-related groups (DRG), (ii) deliveries in which the newborn could not be linked to the mother, (iii) deliveries of mothers younger than 18 or older than 45 years at the delivery, (iv) deliveries before the 22th week or after the 42th week, and (v) deliveries with a lack of information concerning the mode of conception or the potential presence of major defects. Given our interest in investigating the rate of birth defects associated to IVF or ICSI, we also excluded pregnancies obtained with first-level techniques (i.e., pharmacological ovulation induction and intrauterine insemination (IUI)), and cases reported to be ART but not precisely classified. Cohort selection was performed in February 2022.

Detailed information regarding maternal characteristics, type of pregnancy (i.e., single or multiple), and mode of conception (i.e., natural or after ART) were available for all deliveries in the CedAP. Cases of birth defects were identified exclusively using this database. Information on congenital malformations leading to medical pregnancy termination could not be detected. Diagnosis of congenital abnormalities was coded according to the International Classification of Diseases 9th edition—Clinical Modification (ICD-9-CM), Italian version, including structural abnormalities, biochemical abnormalities, and those that are chromosomal or otherwise genetic. Malformations were assessed by the neonatologist at birth. They were not confirmed by a geneticist. Minor defects were not generally coded, and any defect diagnosed after hospital discharge of the neonate could not be included. The included malformations are listed in Supplemental Table S1. Local antenatal screening programs did not differ between ART and non-ART pregnancies and remained unchanged during the whole study period. Stillbirths as well as live births were included in our analysis.

For the study, Statistical Analysis System Software (version 9.4; SAS Institute, Cary, NC, USA) was used. Descriptive statistics were used to summarize the characteristics of mothers. Differences between women conceiving naturally and those undergoing ART were tested by the Chi-square test. The rate of newborns affected by major defects was calculated among natural pregnancies and among pregnancies achieved after ART. A binomial distribution model was used to determine the 95% CI of proportions. To assess the potential association between the use of ART and the detection of major defects at birth, a logistic regression model was implemented to estimate the odd ratio (OR) and the corresponding 95% CI of major defects among natural and ART pregnancies. We obtained crude OR and adjusted OR allowing for maternal sociodemographic features (i.e., age, nationality, marital status, education, employment, and parity) (model 1). Maternal age was included as a categorical variable (categories: < 30 years, 30–34 years, 35–39 years, ≥ 40 years). A second model adjusted also for the plurality was computed (model 2). Finally, analyses were repeated in strata of type of ART.

## Results

We identified 529,289 deliveries that took place in Lombardy between 1 January 2014 and 31 December 2020 from women beneficiaries of NHS. We excluded 3898 records because they did not match to a hospital ICD-9-CM code or to a DRG code related to childbirth, 3086 records related to mothers aged less than 18 or more than 45, 299 records because the gestational age was too short (< 22 weeks) or too long (> 42 weeks), 7626 records because the infant could not be linked to the mother because of a missing identification code, 2063 records because of a lack of information concerning the mode of conception or the potential presence of major defects, 1977 records because related to pregnancies obtained with first level techniques and 1919 because the ART modality was not specified. Overall, a total of 508,421 live births were included, of which 14,067 (2.8%) were achieved after IVF-ICSI. The number of cases per year progressively increased over the years, being 2.1%, 2.3%, 2.6%, 2.8%, 3.2%, 3.4%, and 3.2% in 2014, 2015, 2016, 2017, 2018, 2019, and 2020, respectively (*p* < 0.0001).

The baseline characteristics of natural and ART births are shown in Table 1. Women undergoing ART were more often older, nulliparous, Italian, married, employed, and more highly educated. Multiple and preterm births were also more frequent. The proportion of stillbirth was below 1% in both groups, but slightly higher among ART newborns.

**Table 1:** Selected covariates at baseline in the cohort of natural births and births after Assisted Reproductive Techniques (ART)

Covariates	Natural births		ART births		*p*
	N.	%	N.	%	
Maternal age (years)					
< 30	153,447	31.0	875	6.2	< 0.0001
30–34	172,888	35.0	3488	24.8	
35–39	130,068	26.3	6053	43.1	
≥ 40	37,951	7.7	3641	25.9	
Maternal citizenship					
Italian	347,174	70.2	12,031	85.5	< 0.0001
Not Italian	147,18	29.8	2036	14.5	
Marital status					
Married	314,142	63.9	10,252	73.3	< 0.0001
Not married	177,302	36.1	3733	26.7	
Maternal education					
Middle school	117,087	23.7	1670	11.9	< 0.0001
High school	209,995	42.5	5490	39.0	
University	166,629	33.8	6897	49.1	
Maternal employment					
Employed	319,221	64.7	11,643	82.8	< 0.0001
Not employed	174,511	35.3	2411	17.2	
Parity (number of previous births)					
0	237,453	48.0	11,646	82.8	< 0.0001
≥ 1	256,901	52.0	2421	17.2	
Type of pregnancy					
Singletons	488,234	98.8	12,117	86.1	< 0.0001
Twins	6120	1.2	1950	13.9	
Gestational age at birth (weeks)					
≥ 37	465,079	94.1	11,866	84.4	< 0.0001
< 37	29,275	5.9	2201	15.6	
Vitality status of newborn					
Livebirth	493,034	99.7	14,006	99.6	0.0018
Stillbirth	29,275	0.3	61	0.4	

**Fig. 1:**
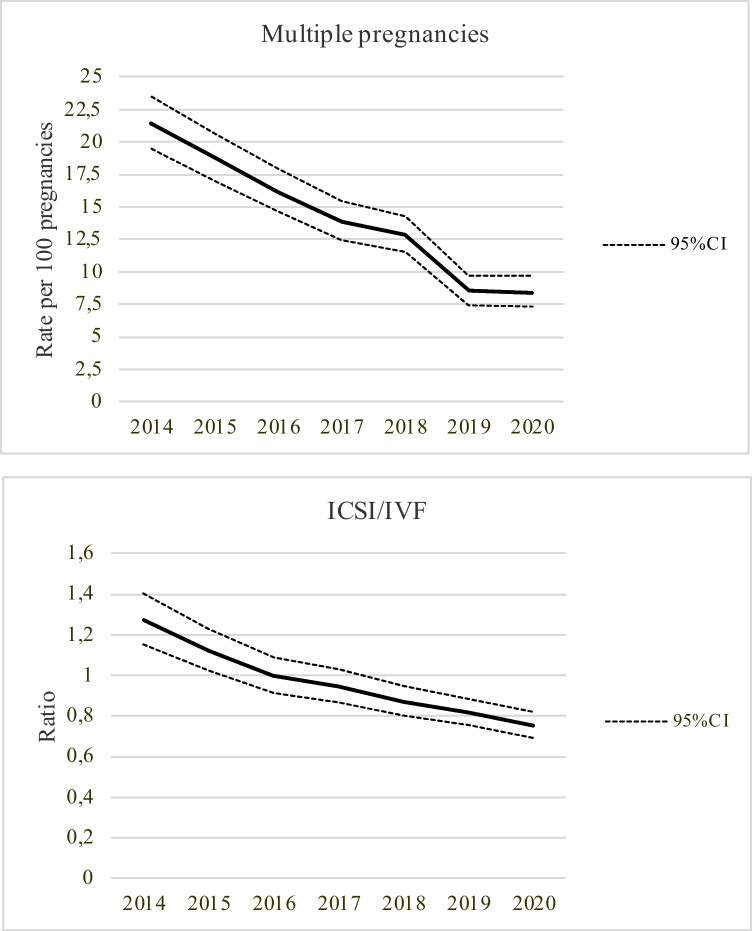
Temporal trends of multiple pregnancies after IVF-ICSI in Lombardy, 2014-2020 (*upper panel*) and of the relative ratio of ICSI over conventional IVF (*lower panel*)

Among ART pregnancies, conventional IVF and ICSI were used in 7252 (51.6%) and 6815 (48.4%) women, respectively. Since multiple pregnancies and ICSI may increase the risk of birth defects, we evaluated whether these two variables changed over time. A statistically significant reduction was observed for multiple pregnancies after ART (*p*-value for trend < 0.0001) (Fig. 1, *upper panel*). In addition, the relative proportion of ART babied born after ICSI decreases. The ratio between ICSI and conventional IVF shrunk over time (*p*-value for trend < 0.0001) (Fig. 1, *lower panel*). Conversely, maternal age did not vary significantly over time (Supplemental Table S2).

Overall, 13,080 birth defects were recorded, of whom 412 occurred among ART newborns (3.1%). The crude OR of birth defects was 1.15 (95% CI: 1.04–1.27), the adjusted OR was 1.09 (95% CI: 0.98–1.21). Data was analyzed separately also for conventional IVF and ICSI. The adjusted ORs were 1.01 (95% CI: 0.88–1.17) and 1.21 (95% CI: 1.01–1.34), respectively.

**Fig. 2:**
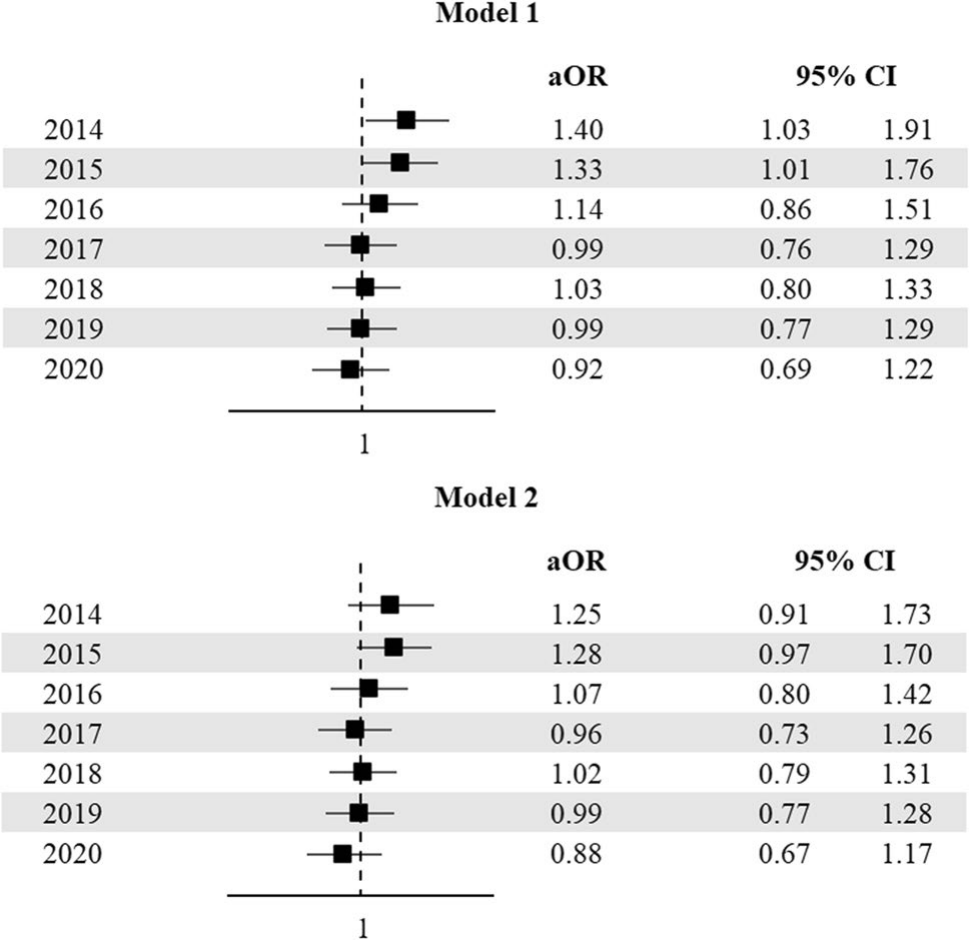
Adjusted odds ratios (aOR) of birth defects according to mode of conception over calendar period. Lombardy, 2014–2020. Model 1: OR adjusted for age, nationality, marital status, education, employment, and parity (*upper panel*). Model 2: OR adjusted for age, nationality, marital status, education, employment, parity, and plurality (*lower panel*)

The details on the birth defects over time are presented in Table 2. The adjusted ORs of birth defects according to the mode of conception over the study period are shown in Fig. 2. While ART was initially related to an increased risk of malformations and the adjusted OR was 1.40 (95% CI: 1.03–1.91) in 2014, no statistically significant associations emerged in the last years, the adjusted OR being 0.92 (95% CI: 0.69–1.22) in 2020 (Fig. 2, *upper panel*, model 1). When we adjusted the model for plurality, the association between ART and birth defects was not statistically significant over the entire period of study (Fig. 2, *lower panel*, model 2).

**Table 2:** Number and rate of birth defects over time according to the mode of conception

Year of birth	Number of births		Number of birth defects		% of birth defects	
	Natural	ART	Natural	ART	Natural	ART
2014	78,704	1650	1565	45	2.0	2.7
2015	76,123	1788	1759	58	2.3	3.2
2016	72,907	1953	1796	56	2.5	2.9
2017	71,276	2079	2092	62	2.9	3.0
2018	68,414	2232	2000	70	2.9	3.1
2019	65,467	2309	1847	66	2.8	2.9
2020	61,463	2056	1609	55	2.6	2.7

Finally, we evaluated separately the trends in IVF and ICSI pregnancies and failed to highlight marked differences in the trends (Supplemental Table S3). The 95% CI was, however, larger.

## Discussion

This population-based study confirmed that the risk of birth defects is increased in women undergoing ART, but also highlighted a decreasing trend over time. Indeed, the adjusted OR dropped from 1.40 (95% CI: 1.03–1.91) in 2014 to 0.92 (95%CI: 0.69–1.22) in 2020 (Fig. 2). Moreover, our data showed a downward trend in both the number of multiple pregnancies and ICSI/IVF ratio over the years.

During the last decades, several studies have shown an increased risk of birth defects after ART [12, 22, 23]. The most recent systematic review and meta-analysis including 46 studies suggested a 40% increased risk (RR: 1.40; 95% CI 1.31–1.49) when considering all procedures together [4]. The impact was milder when considering separately data from conventional IVF or ICSI, presumably because of differences in the quality of the included studies for these two secondary analyses [4]. The RRs were 1.25 (95% CI: 1.12–1.40) and 1.29 (95% CI: 1.14–1.45), respectively.

The mechanisms underlying the association of ART and birth defects are unclear. Possible explanations include a detrimental effect of ovarian hyperstimulation, in vitro culture conditions, freezing-thawing of the embryo, and luteal phase support [4]. Moreover, as mentioned earlier, an intrinsic increased risk in the infertile population is plausible and can explain at least in part this increase.

Although evidence from our study is generally consistent with previous studies (we also highlighted an increased risk), the magnitude of the association is milder, the overall adjusted OR being only 1.19 (95% CI: 0.98–1.21). Of utmost relevance here is the progressive reduction of this association during the 7 years period. This further supports the idea that some important and beneficial changes are ongoing, thus questioning the validity of results obtained from meta-analyses that included studies of pregnancies obtained several years ago. The reduction in birth defects may be driven by the advancement and changes of ART techniques and policies. Previous publications may not reflect the conditions of more recent IVF-ICSI settings. In fact, most studies included in the meta-analyses were performed more than 10 years ago. On the other hand, a previous study aimed at detecting changes in the rate of malformation over time failed to highlight any change [18]. The authors analyzed the hazard of congenital malformations in four time periods (1988–1992, 1993–1997, 1998–2002, and 2003–2007), but failed to show any impact. We attributed this contrasting finding to the studied historical period that radically differed from ours (we focused on 2014–2020).

In our opinion, the most striking determinant of the reduction in the risk of birth defects in our study is the parallel reduction in multiple births (as shown in the upper panel of Fig. 1). In fact, the reduction in twins after ART may have contributed to the normalization of the risk of birth defects in non-spontaneous pregnancies. The observation that, in our study, the trend of reduction over time was tempered with the application of the logistic model 2 (that included multiple pregnancy) supports this view. The reduction in multiple pregnancies is consequent to the progressive diffusion of single embryo transfer, as recommended by international guidelines [24]. Multiple births explain 36% of the relative effect of ART on non-chromosomal birth defects [8].

Other factors may have contributed to the improvement of the rate of birth defects. Firstly, the downward trend in ICSI procedure over the years may deserve attention (Fig. 1, *lower panel*). Noteworthy, the reduction in ICSI use in our analysis contrasts with international trends [25, 26]. This trend may be due to the local context. Of relevance here is that, in Lombardy, all ART units meet at least once a year to discuss clinical issues of common interest since 2011. The abuse of ICSI was repeatedly discussed in these meetings and a position against its systematic and blind use was shared. Of relevance is that, even if not conclusive, there is evidence of an association between birth defects and ICSI compared to conventional IVF [11]. However, a frank increased risk emerged only for genitourinary malformations, the common RR in meta-analyses being 1.27 (95% CI: 1.02–1.59) [13]. The specificity of this finding questions causal relation. A possible genetic or metabolic disturbance causing male infertility in the father and genitourinary malformation in the offspring seems more plausible. Thus, even if a reduction in the rate of ICSI is another encouraging change currently ongoing in the ART field, a potential positive effect on the reduction of the rate of birth defects is unlikely. Our data did not allow us to draw robust information on this issue because the statistical power of the analyses dropped when separating IVF and ICSI pregnancies (Supplemental Table 3).

Secondly, IVF-ICSI procedures are now used to treat a border range of infertile couples, and the treated populations may have slightly changed over time. This is indirectly supported by the growing proportion of ART newborns in Western countries. In our study, we also observed a progressive increase from 2.1% in 2014 to 3.2% in 2020. The most plausible explanation of this increase is the widespread diffusion of IVF-ICSI rather than an increase in the proportion of infertile women. One may even speculate that couples may be treated earlier compared to the past, thus enhancing the proportion of subfertile (rather than infertile) cases. If so, the above-mentioned infertility-related impact on the rate of birth defects could be diluted.

Some limitations of our study should be recognized. The diagnosis of malformation is based on information available at the time of delivery. Thus, no data are available on malformations that can be diagnosed during the infant’s hospitalization until discharge, or later in the lifetime. One cannot exclude that malformations detected later in life could be differently distributed between ART and non-ART newborns. Moreover, we did not also report on minor defects. Furthermore, we have no information on pregnancies terminated early because of birth defects that could be expected to be the most severe. On the other hand, it must be underlined that the mode of assessment was not influenced by the modality of conception. One may infer that this bias may similarly affect natural and ART-mediated conceptions A second limitation is the reliability and completeness of the collected information. Data was collected from registries (as for most of the available evidence) and this modality is known to be exposed to some inaccuracies. Moreover, we did not have information on the indication for ART and details of the procedure. Given the relevance of infertility diagnosis in influencing the rate of malformation, data on the cause of infertility would have been of utmost interest. Collected information did not include the mode of semen collection, i.e., whether it was ejaculated or surgically retrieved, and whether ART was performed with donated gametes. However, these two aspects cannot be expected to play a role in explaining the observed results given their very marginal role in ART during the studied period (https://www.iss.it/web/guest/rpma-dati-registro). Finally, as shown in Table 1, mothers conceiving with or without ART differed for several baseline characteristics. Even if we included these variables in the multivariate model, we cannot exclude residual confounders. On the other hand, it should be highlighted that the screening program for ART and non-ART pregnancies did not differ in Lombardy and did not change overtime during the studied period. Among the strengths of this study, we should consider the population-based design and the large sample size. Moreover, to our knowledge, this is the first contribution from Southern Europe and refers to a recent historical period (2014–2020).

In conclusion, our study shows that the association between ART and birth defects is not steady over time. In our context, we observed a reduction of the risk during the study period. Further studies focusing on different areas are needed to confirm the presence of this improvement. Evidence obtained more than one decade ago may not be valid in the modern era of IVF.

## Supplementary information

ESM 1 (DOCX 16 kb)

ESM 2 (DOCX 14 kb)

ESM 3 (DOCX 13 kb)
