# Supplementary material for: Trends in the incidence of major birth defects after assisted reproductive technologies in Lombardy Region, Northern Italy
Source: J Assist Reprod Genet. 2023 Feb 10;40(4):857–63. doi: 10.1007/s10815-023-02732-z (PMC10224879; doi:10.1007/s10815-023-02732-z)
Supplement: Supplementary file 1 — (DOCX 16 kb) [file 10815_2023_2732_MOESM1_ESM.docx]

| **Table S1**. List of major defects considered. | | | |
| --- | --- | --- | --- |
|  | | | |
| ICD-9-CM code | Type of defect | Cases | |
|  |  | N | % |
| 740.X | Anencephaly | 9 | 0.1 |
| 741.XX | Spina bifida | 32 | 0.2 |
| 742.XX | Other congenital anomalies of the nervous system (e.g., encephalocele, microcephaly, congenital hydrocephalus, etc.) | 455 | 3.5 |
| 743.XX | Congenital anomalies of the eye (e.g.; anophthalmia, microphthalmia, buphthalmosetc.) | 64 | 0.5 |
| 744.XX | Congenital anomalies of the ear, face and neck | 177 | 1.4 |
| 745.XX | Abnormalities of the bulb of the heart and anomalies of cardiac septal closure (e.g., ventricular septal defect, double outlet of the right ventricle, ostium secund atrial septal defect,etc.) | 2,944 | 22.5 |
| 746.XX | Other congenital anomalies of the heart | 289 | 2.2 |
| 747.XX | Other congenital anomalies of the circulatory system | 1,269 | 9.7 |
| 748.XX | Congenital anomalies of the respiratory system (e.g, choanal atresia, nose absent, absence or agenesis of bronchi, larynx or trachea, etc.) | 151 | 1.2 |
| 749.XX | Cleft palate and cleft lip | 380 | 2.9 |
| 750.XX | Other congenital anomalies of the alimentary superior tract (e.g., macroglossia, congenital hiatus hernia, etc.) | 1,409 | 10.8 |
| 751.XX | Other congenital anomalies of the digestive system (e.g., Meckel's diverticulum, atresia and stenosis of small intestine, etc.) | 255 | 1.9 |
| 752.XX | Congenital anomalies of the genital organs (e.g., hypospadias, pure gonadal dysgenesis, embryonic cyst of the fallopian tubes and broad ligaments, etc.) | 1,555 | 11.9 |
| 753.XX | Congenital anomalies of the urinary system (e.g., agenesis and dysgenesis of the kidney, cystic kidney disease, multicystic kidney, etc.) | 974 | 7.4 |
| 754.XX | Congenital malformations of the system skeletal muscle (e.g., flatfoot, clubfoot, congenital dislocation of the hip, etc.) | 1,061 | 8.1 |
| 755.XX | Other congenital limb anomalies (e.g., polydactyly, syndactyly, etc.) | 730 | 1.8 |
| 756.XX | Other congenital musculoskeletal anomalies (e.g., absence of skull bones, abnormalities of the diaphragm, spina bifida occulta, etc.) | 397 | 3.0 |
| 757.XX | Congenital anomalies of the integument | 195 | 1.5 |
| 758.XX | Chromosomal abnormalities (e.g., Down syndrome, Turner syndrome, Klinefelter syndrome, etc.) | 418 | 3.2 |
| 758.XX | Other congenital anomalies, unspecified | 154 | 1.3 |
| Codes not related to congenital anomalies or missing | Not defined | 162 | 1.2 |
