# Supplementary material for: Trends in the incidence of major birth defects after assisted reproductive technologies in Lombardy Region, Northern Italy
Source: J Assist Reprod Genet. 2023 Feb 10;40(4):857–63. doi: 10.1007/s10815-023-02732-z (PMC10224879; doi:10.1007/s10815-023-02732-z)
Supplement: Supplementary file 2 — (DOCX 14 kb) [file 10815_2023_2732_MOESM2_ESM.docx]

| **Table S2**. Maternal age at birth of women undergoing Assisted Reproductive Techniques (ART) over time. | | | | | | | |
| --- | --- | --- | --- | --- | --- | --- | --- |
|  | | | | | | | |
|  | Calendar year | | | | | | |
| Maternal age (years) | 2014 | 2015 | 2016 | 2017 | 2018 | 2019 | 2020 |
| <30 | 107 (6.5) | 95 (5.3) | 124 (6.4) | 116 (5.6) | 176 (7.9) | 119 (5.2) | 138 (6.7) |
| 30-34 | 445 (27.0) | 455 (25.5) | 474 (24.3) | 519 (25.0) | 514 (23.0) | 576 (25.0) | 505 (24.6) |
| 35-39 | 689 (41.8) | 782 (43.7) | 833 (42.7) | 886 (42.6) | 968 (43.4) | 1,001 (43.4) | 894 (43.5) |
| ≥40 | 409 (24.8) | 456 (25.5) | 522 (26.7) | 558 (26.8) | 574 (25.7) | 613 (26.6) | 519 (25.2) |
