# Supplementary material for: Trends in the incidence of major birth defects after assisted reproductive technologies in Lombardy Region, Northern Italy
Source: J Assist Reprod Genet. 2023 Feb 10;40(4):857–63. doi: 10.1007/s10815-023-02732-z (PMC10224879; doi:10.1007/s10815-023-02732-z)
Supplement: Supplementary file 3 — (DOCX 13 kb) [file 10815_2023_2732_MOESM3_ESM.docx]

**Supplemental Table S3:** Adjusted ORs over time in conventional IVF and ICSI pregnancies separately.

| Study year | Conventional IVF | ICSI |
| --- | --- | --- |
| 2014 | 1.35 (0.85-2.14) | 1.44 (0.96-2.16) |
| 2015 | 1.33 (0.90-1.97) | 1.33 (0.92-1.93) |
| 2016 | 0.81 (0.52-1.27) | 1.48 (1.04-2.09) |
| 2017 | 0.88 (0.60-1.29) | 1.10 (0.77-1.57) |
| 2018 | 0.84 (0.58-1.21) | 1.25 (0.90-1.74) |
| 2019 | 1.08 (0.78-1.50) | 0.89 (0.60-1.32) |
| 2020 | 1.02 (0.72-1.44) | 0.80 (0.51-1.25) |

ORs were adjusted for age, nationality, marital status, education, employment, parity, and plurality.
